# Fetoscopic three-port, three-layer open spina bifida repair

**DOI:** 10.3171/2026.1.FOCVID25230

**Published:** 2026-04-01

**Authors:** Vaidehi Mahadev, Rya Muller, Amir Alhajjat, Aimen F. Shaaban, Robin Bowman

**Affiliations:** Divisions of 1 Pediatric Neurosurgery and; 2 Pediatric Surgery, The Chicago Institute for Fetal Health, Ann & Robert H. Lurie Children’s Hospital of Chicago, Northwestern University Feinberg School of Medicine, Chicago, Illinois

**Keywords:** open spina bifida, myelomeningocele, fetoscopic closure, prenatal surgery, fetal surgery

## Abstract

Prenatal surgery for open spina bifida has demonstrated improved neurological and functional outcomes compared to postnatal closure. However, open fetal surgery is associated with increased obstetric complications. Minimally invasive fetoscopic approaches were developed to decrease maternal morbidity from large hysterotomies while preserving fetal benefits. Single- and two-layer fetoscopic closure techniques have been described but there is concern for the ability to achieve watertight closure. Watertight closure is critical for preventing postoperative complications, particularly cerebrospinal fluid leakage and wound dehiscence. This video describes a case presentation for a novel three-port, three-layer fetoscopic repair modeled after a postnatal closure technique.

The video can be found here: https://stream.cadmore.media/r10.3171/2026.1.FOCVID25230

## Transcript

### 0:27 Clinical Presentation.

This is a case presentation of a fetoscopic closure of a myelomeningocele. This mother is a 30-year-old G2P0 woman who was referred at 21 and 1 weeks gestation to our institution with a singleton pregnancy complicated by an open neural tube defect. Her 19-week ultrasound demonstrated an L3 open spina bifida, normal ventricular size at 9 mm bilaterally, and a Chiari II malformation.

### 0:55

This ultrasonic video demonstrates axial views through the open spina bifida. The neural placode ascends through the bifid spine toward the dorsum of the myelomeningocele. Amniocentesis was completed and confirmed a normal karyotype with acetylcholinesterase present. Fetal echocardiogram was normal. The patient was referred to our fetal center for consultation. The patient underwent extensive counseling and was determined to be a fetal surgery candidate based on our institutional criteria.^1^ She elected to proceed with fetoscopic in utero closure of the open defect.

### 1:32

Fetal MRI at 21 weeks confirmed the lumbosacral myelomeningocele, hindbrain herniation, and normal ventricular size. There was good movement at the hips, knees, and ankles and no foot deformity was noted.

### 1:47 Surgical Procedure.

The uterus is approached by a midline laparotomy. An ultrasound is used to map the location of the placenta and major vessels on the surface of the uterus. Under ultrasonic guidance, four full-thickness sutures are placed through the uterine wall to delineate a 1-cm square area through which a 10-French trocar is inserted using Seldinger technique. After removing a portion of the amniotic fluid, the uterus is insufflated with warm humidified carbon dioxide. Two subsequent trocars are placed under fetoscopic visualization. Prior to surgical intervention, the fetus is administered a cocktail of rocuronium, fentanyl, and atropine.

### 2:33

We initially see the large myelomeningocele. Using sharp dissection, we enter into the arachnoid starting inferolaterally. Looking within the meningocele sac, one can appreciate the nerve roots on the ventral aspect of the placode, which are on stretch given the extreme size of the meningocele sac. Circumferentially, the arachnoid is incised, being careful to avoid injury to the spinal cord and lateral dorsal roots, as well as the segmental vasculature. The neural placode is imbricated with an interrupted 6-0 nonabsorbable monofilament suture.

### 3:16

The dura, attached laterally and ventrally to the open skin edge at the junctional zone, is identified at its lateral extent and detached. The dura is medialized by gently freeing it from the underlying fascia. In cases with insufficient robust dura, either a myofascial flap or dural substitute may be utilized to achieve a watertight dural closure. A running nonabsorbable 6-0 monofilament suture is used to reapproximate the delicate dura.

### 3:49

Skin closure is performed in the midsagittal plane by mobilizing the skin and subcutaneous fat layer. For defects too large for primary skin closure, an acellular dermal matrix may be sutured to the edges of healthy skin. The ports are removed, and the insertion sites are closed with an absorbable suture. The uterus is returned to the abdominal cavity, and the laparotomy is closed in the standard fashion.

### 4:18 Postoperative Course—Immediate.

Postoperative fetal MRI completed at 30 weeks’ gestation demonstrates the improvement in the hindbrain herniation with the repaired myelomeningocele site and a stable ventricular size.

### 4:30

The child was born at 36 weeks via vaginal delivery. The back wound was well healed with no evidence of CSF leak or other concerns. He did well following birth with muscle strength testing by physical therapy demonstrating good strength throughout his lower extremities.

### 4:49

He had no evidence of hydrocephalus or brainstem dysfunction with good coordination of breathing, sucking, and swallowing. His bladder was managed according to the CDC UMPIRE urological protocol^2^ with catheterization discontinued on day-of-life 3. His hospital course was complicated by a urinary tract infection requiring IV antibiotics.

### 5:13 Postoperative Course—Long-Term.

The child is followed in our multidisciplinary spina bifida center by pediatric neurosurgery, urology, orthopedics and rehab medicine. He remains without clinical evidence of hydrocephalus or Chiari II symptoms. His yearly muscle strength testing by physical therapy has demonstrated no decline. He ambulated independently at 17 months and toilet trained by 3 years of age. His urodynamic study demonstrates safe function despite a small capacity.

### 5:45

Routine imaging has demonstrated a mild to moderate ventricular size with resolution of the hindbrain herniation given the lack of tonsillar descent and the presence of the fourth ventricle. He has a low-lying spinal cord as expected for a child with a history of open spina bifida.

### 6:04 Discussion.

The MOMS trial revolutionized treatment of open spina bifida as it demonstrated enhanced strength in the lower extremities, reduced hindbrain herniation, and less need for hydrocephalus treatment.^1^

### 6:17

The open uterine surgical technique requires a large hysterotomy, which is associated with increased maternal risk, including an increased risk of spontaneous uterine rupture with the potential for fetal loss in subsequent pregnancies.^1,3,4^ Fetoscopic myelomeningocele repair allows for spina bifida closure in utero while reducing the risk to the mother. This technique avoids the large hysterotomy.^5^ It allows the opportunity for vaginal delivery, effectively eliminating the additional risk of uterine rupture.^5^ Kohn et al. showed that 50% of patients were able to undergo vaginal delivery following fetoscopic surgery, compared to none of those who have undergone open uterine fetal surgery.^6^

### 7:07

In the ideal situation, the fetus’s back may be closed primarily. When there is insufficient skin for a primary closure, a dermal matrix substitute may be utilized to bridge the skin gap. This substitute will be present at the time of birth, as demonstrated here. This area should be kept clean and moist as the infant re-epithelializes this area.^7^

### 7:32

In the original MOMS trial, the closure technique required at least one layer, dura only or dura and fascia, followed by skin closure.^1,8^ However, some degree of variability existed in closure techniques among the three participating centers.^1,8^ In prior reports of fetoscopic myelomeningocele repair, failure to achieve a watertight dural closure is likely related to technical compromises of the single-layer or two-layer closure.^9,10^ Research has demonstrated that three-layer closures reduce the risk of CSF leak and the need for wound revisions in comparison to single-layer closures.^9,10^

### 8:14

In conclusion, prenatal surgery for open spina bifida has demonstrated improved neurological and functional outcomes compared to postnatal closures. However, open maternal fetal surgery is associated with increased obstetrical complications, such as uterine ruptures and the need for cesarean deliveries in the current and subsequent pregnancies.^1,3,4^ Fetoscopic approaches decrease maternal morbidity from large hysterotomies while preserving the fetal benefits of prenatal repair.^5^ Watertight dural closure is critical for preventing postoperative complications such as CSF leak and wound dehiscence.^10^ In this video we present a minimally invasive uterine approach using a three-port, three-layer fetoscopic myelomeningocele closure modeled after our postnatal closure technique. This approach presents less risk to the mother while preserving the meaningful neurological outcomes for the child.

## Disclosures

Dr. Bowman reported grants from Kiwanis Neuroscience Research Foundation during the conduct of the study.

## Author Contributions

Primary surgeon: Bowman. Assistant surgeon: Mahadev, Alhajjat, Shaaban. Editing and drafting the video and abstract: Bowman, Muller, Shaaban. Critically revising the work: Bowman, Muller, Shaaban. Reviewed submitted version of the work: Bowman, Muller, Alhajjat, Shaaban. Approved the final version of the work on behalf of all authors: Bowman. Supervision: Bowman.

## Supplemental Information

### Patient Informed Consent

The necessary patient informed consent was obtained in this study.
